# Anatomical and imaging measurements of the angle between the axis of the lumbar pedicle and lateral isthmus margin and its clinical significance

**DOI:** 10.1186/s13018-023-03983-3

**Published:** 2023-07-18

**Authors:** Shuiquan Wang, Dilimulati Aikeremu, Alafate Kahaer, Abulikemu Maimaiti, Yang Xiao, Abudusalamu Tuoheti, Rui Zhang, Xieraili Maimaiti, Hailong Guo, Paerhati Rexiti

**Affiliations:** 1 Department of Anatomy, College of Basic Medicine, Xinjiang Medical University, Urumqi, China; 2 Department of 2nd Spine Surgery, People’s Hospital of Xinjiang, Urumqi, China; 3 Departments of Spine Surgery, The First Affiliated Hospital of Xinjiang Medical University, Urumqi, China

**Keywords:** Pedicle axis, Lateral margin of isthmus, Angle, Pedicle screw

## Abstract

**Background:**

This study aims to explore the measurement of the angle between the axis of the pedicle and the lateral margin of the isthmus on the lumbar spine, and investigate its clinical significance.

**Methods:**

The angle was measured on 120 normal adults’ X-ray and 25 dry anatomical specimens. 60 screws were placed by junior residents on 6 wet specimens through the freehand technique. 30 screws were placed on one side with their original experience. After learning the techniques mentioned in the study, 30 screws were placed on the other side. The specimens were examined by X-ray and CT, and the angles of the screw paths and the integrity of the pedicle were evaluated.

**Results:**

The angles of 120 subjects and 25 anatomical specimens show a gradually increasing trend. The differences among each segment were statistically significant (*P* < 0.05), but the difference in the same segment between the X-ray and the anatomical specimens was not statistically significant (*P* > 0.05). Furthermore, the differences in L1, L2, and L3 between the two genders were not statistically significant (*P* > 0.05). However, the angles were larger in female group than in male group in L4 and L5, and the differences were statistically significant (*P* < 0.05). The difference in the deviation rate of screw placement before and after the learning was statistically significant only in the L5 segment (*P* < 0.05). The difference in overall excellence rate was statistically significant (*P* < 0.05).

**Conclusions:**

The measurement of the angle between the axis of the pedicle and the lateral margin of the isthmus on the lumbar can improve the accuracy of the lumbar sagittal screw angle.

## Background

The error of the sagittal screw angle (SSA) of the pedicle is one of the risk factors. To reduce complications, repeated fluoroscopy is often used to improve the accuracy. How to reduce intraoperative radiation and improve safety through the inherent anatomic markers of the spine has been the goal of orthopedists [1–4]. At present, Xiao et al. [5] reported that the SSA could be determined through the included angle between the lateral margin of the lumbar lamina and the axis of pedicle. However, there is no related report on the determination of SSA through the angle between the isthmus and the axis of the pedicle. The author considers it can be used as a reliable reference for spine surgery.

## Materials and methods

### Acquisition of 3D reconstruction data

#### Source of the subjects

A total of 362 anterior–posterior and lateral X-rays of normal lumbar vertebrae were collected from the Imaging Center of the First Affiliated Hospital of Xinjiang Medical University. Twenty-five dry specimens of the lumbar vertebrae with the complete structure were selected (Provided by the Anatomy Teaching and Research Office of Xinjiang Medical University). Six wet specimens of the lumbar with a complete structure (3 men and 3 women).

#### Inclusion and exclusion criteria

Inclusion criteria: (1) The X-ray with good quality and the structures were displayed apparently; (2) Within the age of 20–50 years; (3) Gender equality. Exclusion criteria: (1) The X-rays with inferior quality; (2) Lumbar sacralization or lumbarization. 120 cases (Male: 60, female: 60) were selected according to the inclusion criteria. Each gender group was divided into 5 subgroups, according to five different segments of the lumbar vertebrae.

#### Experimental instruments and materials

1. A protractor (accuracy of 1°, estimate of 0.1°).
2. A medical electric drill, and 1-mm, 1.5 mm, and 2-mm Kirschner wires.
3. Titanium alloy pedicle screws with a diameter of 5.5–6.0 mm, with lengths of 40 and 45 mm (produced by Zhengtian Instrument Co., Ltd., Tianjin, China).
4. Imaging instruments: Hitachi 500 mA DR X-ray machine, Japan; Siemens computed tomography (CT) system, Germany; PACS image software system.

#### Imaging data measurement

**Fig. 1:**
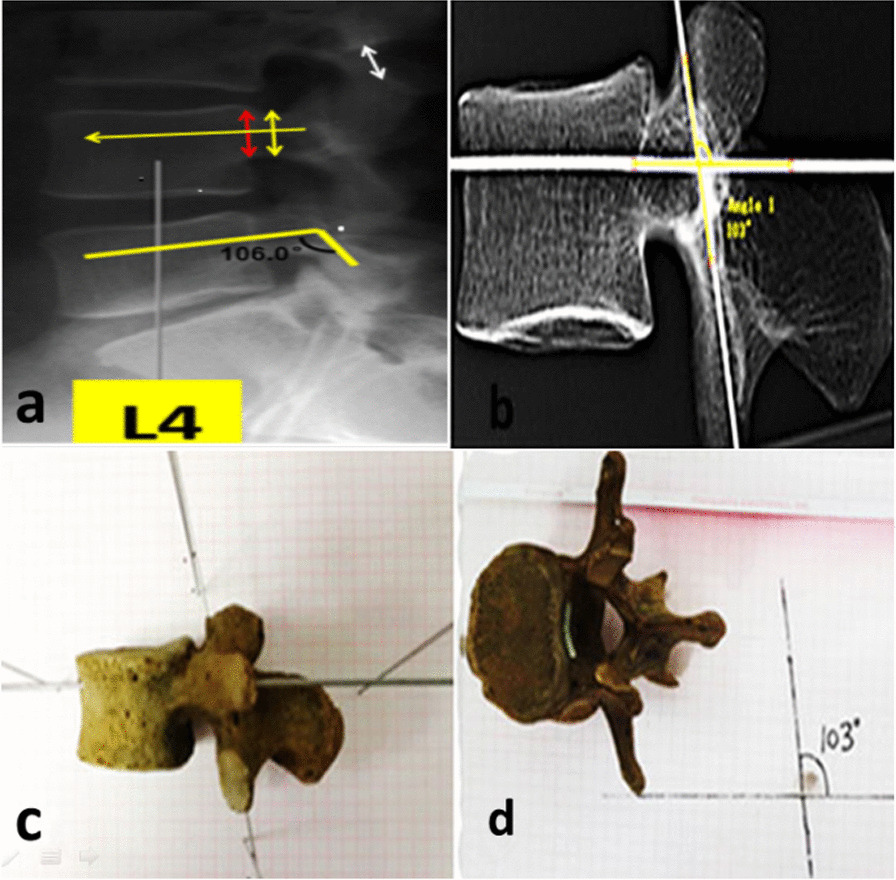
Different measuring methods **a** Measurement of the angle between the axis of the pedicle and the lateral margin of the isthmus; **b**–**d** Imaging and anatomical measurement of the angle between the lumbar isthmus and axis of the lumbar pedicle in the pre-experiment. Kirschner wires were inserted using the Ebraheim method [6], and the angles between these Kirschner wires were measured

It is required that the bilateral pedicles of the pedicle and the upper and lower endplates are completely overlapped, without bilateral shadows (Fig. 1a).

1. *Confirmation of the axis of the pedicle*: The middle point of the narrowest section of the pedicle (short yellow arrow) and the middle point of the junction between the pedicle and the vertebral body (short red arrow) were first determined along the pedicle. The axis is the connecting line between the two midpoints shown above (the mid-long yellow arrow).
2. *Confirmation of the lateral margin of the isthmus*: Because only the surface of the isthmus can be seen during the operation, the center axis of the isthmus cannot match the actual demand. The lateral margin of the isthmus was determined on the surface of the lamina (the line between the lower margin of the superior articular process and the upper margin of the inferior articular process on the same segment). To facilitate the determination of the upper margin of the inferior articular process, the upper margin of the superior articular process of the caudal vertebral body was used as a reference.
3. *Angle measurement*: The angle was measured using the picture archiving and communication system (PACS).

### Measurement of the anatomical specimen

#### Pre-experiment

Two 2.0 mm fine Kirschner wires were inserted into the axis of the pedicle and isthmus through the Ebraheim method [6]. Then, the lateral X-ray was performed, and the angle between two Kirschner wires was measured through the PACS.

In addition, the coordinate projection method was used to measure the same angle between the two Kirschner wires in the specimen. The specimen was placed on a plastic foam board with the lateral side perpendicular to the board. Then, 1.0-mm Kirschner wires were vertically inserted into the foam plate at the upper, lower, left, and right sites along the course of the two Kirschner wires in the specimen. Angles formed by connecting the corresponding holes on the board were measured using protractor after removing the specimen..

The angles between the two different methods mentioned above were compared (Fig. 1b–d). It was found that there was no difference between the results. Subsequent specimens were not measured using the Ebraheim method, but the coordinate projection method was used.

#### Confirmation of the axis of the pedicle

The base of the pedicle is almost vertical to the corresponding vertebral body [7, 8]. The midpoint of the narrowest part of the pedicle was marked. Then, the midpoint of the upper and lower margin of the junction between the pedicle and the posterior wall of the vertebral body was determined, and these two points were connected into a straight line. After the line was drawn, it was confirmed whether the line was vertical to the posterior wall of the vertebral body. The electrocardiogram (ECG) coordinate paper was attached to the foam plastic board, and the specimen was placed on it laterally. The researcher held one Kirschner wire in hand, and placed it over the pedicle and parallel to the surface of the board, instead of inserting these into the bone using an electric drill introduced by Ebraheim [6]. These two marked midpoints of the pedicle and the connecting line were observed, and the Kirschner wires on the hand were finely adjusted to keep parallel to the connecting line and perpendicular to the posterior wall of the vertebral body. The researcher held the Kirschner wire and maintained it still, while the assistant inserted two 1.0-mm fine Kirschner wires along the direction of the Kirschner wire into the plastic foam board covered in coordinate paper and fixed on the same side of the Kirschner that the researcher was holding. With the aid of the small lattices on the coordinate paper, the projection of the axis of the pedicle could be accurately marked on the coordinate paper.Confirmation of the lateral margin of the isthmus was using the same coordinate projection method mentioned above to confirm.

#### Angle measurement

Four small holes and one big hole stamped by the transverse process were left on the coordinate paper. The big hole brang difficulties in confirming the location of the intersection point. To mark the cross point of these two lines, a piece of transparent plastic film was used to cover the coordinate paper. Then, the small holes left by the Kirschner wires were connected in pairs using a pen to form the angles on the plastic film. The left and right angles of the specimen were recorded respectively, and the mean value was calculated.

#### Screw placement by residents

**Fig. 2:**
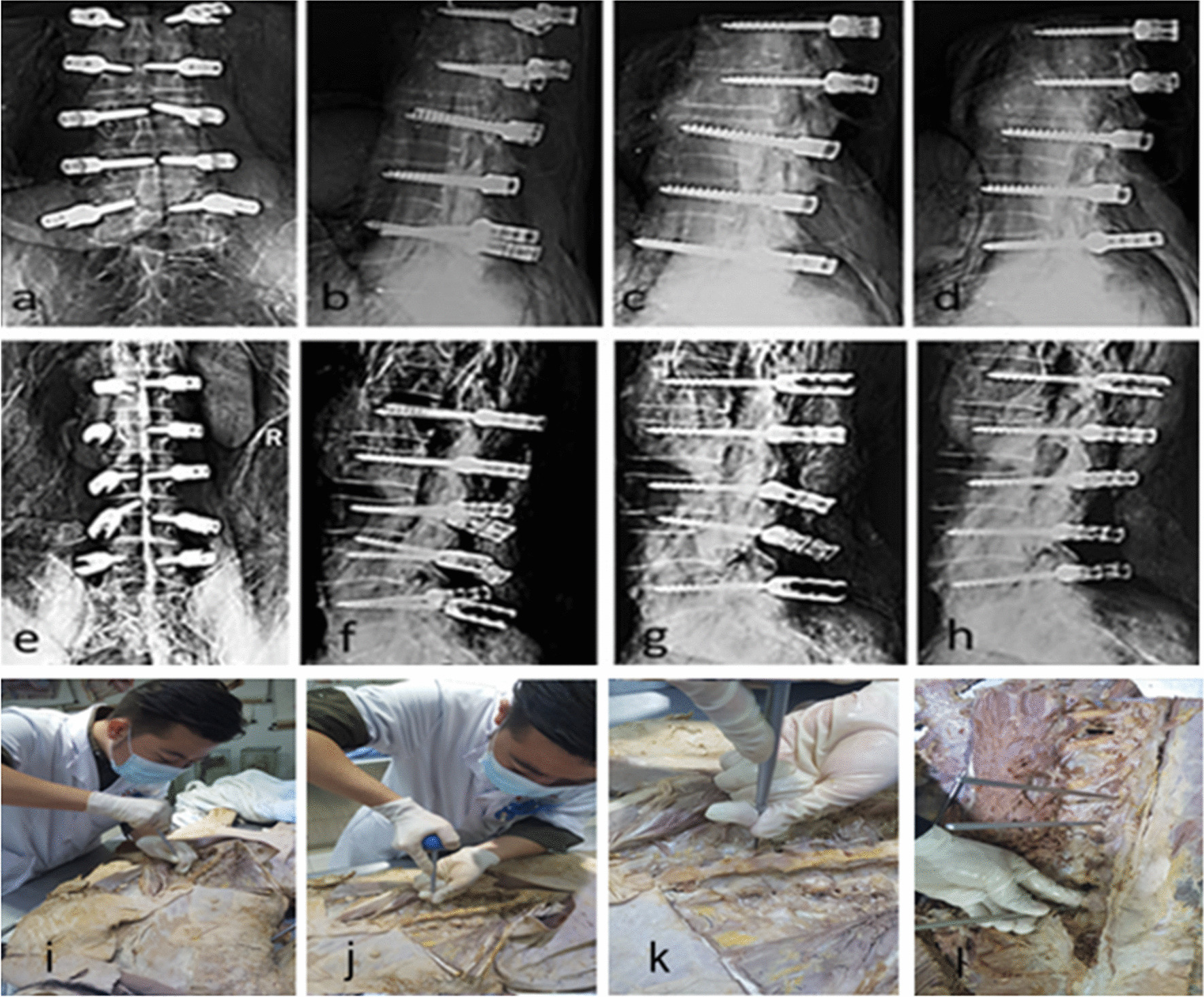
X-ray results of the screw placement specimen 1: **a**–**c** specimen 1 before learning; **d** specimen 1 after learning; **e**–**g** specimen 2 before learning; **h** specimen 2 after learning. **I**–**l** Young resident spine surgeon is operating on wet anatomical specimens

The angle of each segment of the six wet specimens was measured by X-ray before placement, which was used to calculate the deviation rate at last. Operators were randomly selected from the junior resident in the department of spine surgery, who subsequently placed screws without the aid of an X-ray. First, the 30 screws were placed on one side based on their original free-hand experience, and then 30 on the other side referring to the author’s new technique (based on the average value on each segment was calculated on the 120 X-rays and 25 dry specimens) (Fig. 2). Nevertheless, the residents did not know exactly the angles used to calculate the deviation rate. The specimens were examined by X-ray and CT.

**Fig. 3:**
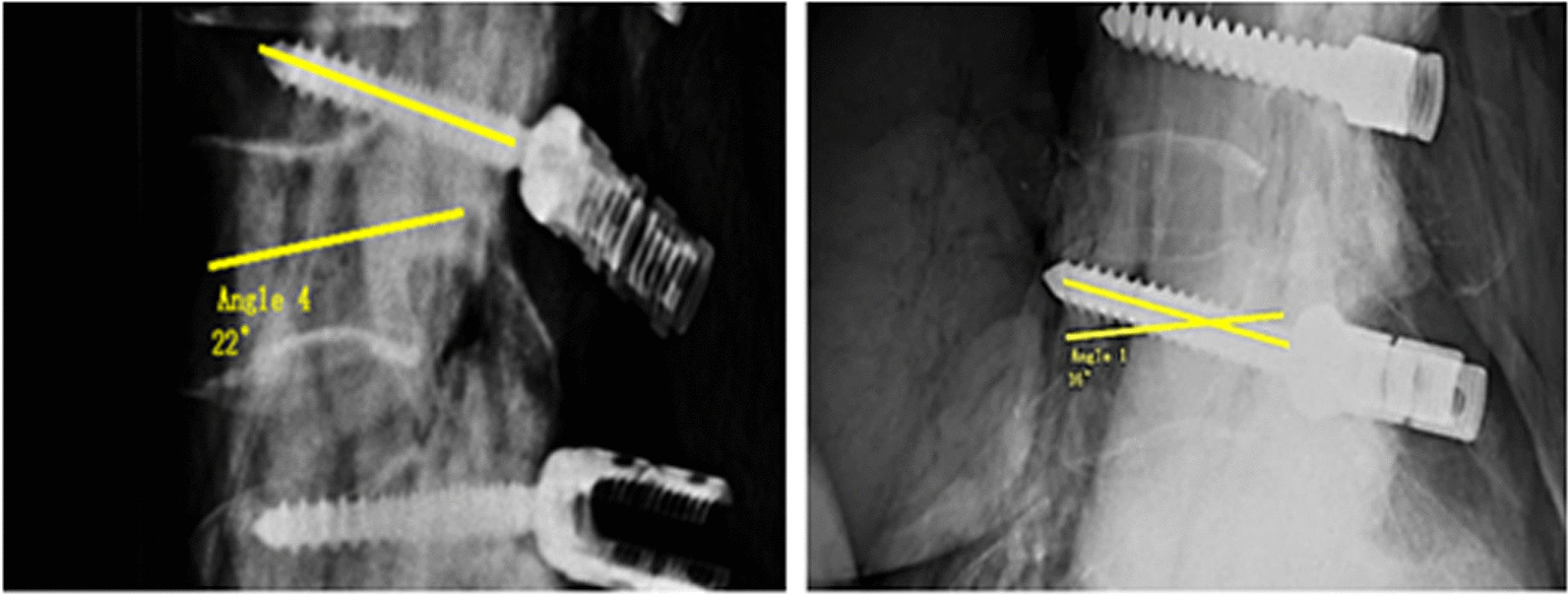
Measurement method for the screw angle

The angle between the center line of the screw and the anatomic axis of the pedicle was defined as the screw angle (SA) (Fig. 3). These were evaluated according to the screw placement criteria proposed by Tiansi Tang 9:(1) Excellent, the screw is within the pedicle, SA is ≤ 5°;(2) Good, within the pedicle, SA is within 5 and 10°; (3) Acceptable, SA is ≥ 11°; (4) Poor, beyond the pedicle cortex or the vertebral body, and enters into the intervertebral space.

In addition, the ratio of the screw angle to the included angle measured by X-ray was defined as the deviation rate: Screw angle/Included angle × 100%.

### Statistical analysis

All data were analyzed using SPSS 18.0. A *T* test was used to compare the difference between specimen and imaging data. The excellence rate of the screw before and after the learning was compared using the Chi-square test. The inspection level was set at *α* = 0.05.

## Results

### Imaging and anatomic specimen measurement results

The results of 120 patients X-ray and 25 dry specimens were demonstrated in Table 1.

**Table 1:** Measurement of X-ray angles and anatomic specimen angles and comparison in different gender group

Vertebral sequence	X-ray			Anatomic Angle (χ̅ ± s, °)	P_1_	t	P_2_
	Male	Female	Mean				
L1	92.5 ± 1.0	92.7 ± 1.1	92.6 ± 1.1	93.0 ± 1.1	0.523	3.334	0.070
L2	93.7 ± 1.6	93.8 ± 1.6	93.7 ± 1.5	94.1 ± 1.1	0.870	1.439	0.232
L3	97.0 ± 1.8	97.4 ± 1.7	97.2 ± 1.8	97.0 ± 1.8	0.290	0.257	0.613
L4	99.3 ± 1.9	100.2 ± 2.5	99.8 ± 2.2	99.6 ± 1.7	0.017*	0.146	0.703
L5	102.7 ± 0.5	105.0 ± 0.7	103.9 ± 1.3	104.0 ± 1.9	0.000*	0.24	0.625

P_1_: Statistical comparison of imaging angles between male and female subjects

P_2_: Statistical comparison of results between imaging and anatomic measurements

*P < 0.05

### Results of screw placement by residents

It revealed that 57 screws were placed within the pedicle. One screw was placed in the intervertebral space, one screw was placed out of the pedicle, and one screw perforated the inner wall of the pedicle. Furthermore, all three failed screws were found in the control group (before learning).

According to the evaluation criteria of the SSA proposed by Tang et al. [9], among the 30 screws in the control group, 17 screws were excellent, 9 screws were good, 1 screw was acceptable, and 3 screws were poor. The excellent rate was 57.08%, and the mean deviation rate was 5.17% (Table 2).

**Table 2:** Comparison of screw placement before and after the learning of resident doctors

Angle of L1 (°)	Control group			Experimental group		
	Screw angle (°)	Classification	Deviation rate (%)	Screw angle (°)	Classification	Deviation rate (%)
L1						
92	3	Excellent	3.26 (3/92)	2	Excellent	2.17 (2/92)
92.5	1	Excellent	1.08 (1/92.5)	1	Excellent	1.08 (1/92.5)
93.5	6	Good	6.42 (6/93.5)	2	Excellent	2.14 (2/93.5)
93	2	Excellent	2.15 (2/93)	1	Excellent	1.08 (1/93)
92.3	2	Excellent	2.17 (2/92.3)	0	Excellent	0
91.8	0	Excellent	0	0	Excellent	0
	Excellent rate	83.30%	Mean 2.51%	Excellent rate	100%	Mean 1.08%
L2						
93	2	Excellent	2.15 (2/93)	2	Excellent	2.15 (2/93)
94	6	Good	6.38 (6/94)	1	Excellent	1.06 (1/94)
93	3	Excellent	3.23 (3/93)	0	Excellent	0
94.3	4	Excellent	4.24 (4/94.3)	2	Excellent	2.12 (2/94.3)
93.5	1	Excellent	1.07 (1/93.5)	1	Excellent	1.07 (1/93.5)
94.2	0	Excellent	0	2	Excellent	2.12 (2/94.2)
	Excellent rate	67.70%	Mean 2.85%	Excellent rate	100%	Mean 1.42%
L3						
96.5	7	Good	7.25 (7/96.5)	2	Excellent	2.07 (2/96.5)
97.5	4	Excellent	4.1 (4/97.5)	0	Excellent	0
97	2	Excellent	2.06 (2/97)	3	Excellent	3.09 (3/97)
97.2	6	Good	6.17 (6/97.2)	5	Excellent	5.14 (5/97.2)
96.4	2	Excellent	2.07 (2/96.4)	2	Excellent	2.07 (2/96.4)
97.9	2	Excellent	2.04 (2/97.9)	2	Excellent	2.04 (2/97.9)
	Excellent rate	67.70%	Mean 3.95%	Excellent rate	100%	Mean 2.40%
L4						
99	4	Excellent	4.04 (4/99)	6	Good	6.06% (6/99)
101	7	Good	6.93 (7/101)	3	Excellent	2.97% (3/101)
100.1	22	Poor	21.98 (22/100.1)	0	Excellent	0
100.3	7	Good	6.98 (7/100.3)	3	Excellent	2.99 (3/100.3)
99.5	3	Excellent	3.02 (3/99.5)	2	Excellent	2.01 (2/99.5)
100	2	Excellent	2 (2/100)	4	Excellent	4 (4/100)
	Excellent rate	50.00%	Mean 7.50%	Excellent rate	83.30%	Mean 3.01%
L5						
103.5	16	Poor	15.46 (16/103.5)	5	Excellent	4.83 (5/103.5)
105.5	5	Excellent	4.74 (5/105.5)	4	Excellent	3.79 (4/105.5)
106	7	Good	6.60 (7/106)	7	Good	6.6 (7/106)
102.5	11	Acceptable	10.73 (11/102.5)	6	Good	5.85 (6/102.5)
103	7	Good	6.8 (7/103)	2	Excellent	1.94 (2/103)
102.8	10	Good	9.73 (10/102.8)	6	Good	5.84 (6/102.8)
	Excellent rate	16.70%	Mean 9.01%	Excellent rate	50%	Mean 4.81%

Among the 30 screws in the experimental group (after learning), 26 screws were excellent, 4 screws were good, and none of the screws was found to be acceptable or poor. The excellent rate was 86.67%, and the mean deviation rate was 2.68% (Table 2).

### Statistical comparison of the results of screw placement by residents

The difference in the deviation rate of screw placement at L1–L4 before and after the learning was not statistically significant, but L5 was significant (*t* = 5.976, *P* < 0.05) (Table 3). After learning, the excellent rate of screw placement was significantly higher (*χ*^2^ = 15.729, *P* < 0.05).

**Table 3:** Comparison of screw placement deviation rates before and after the learning of resident doctors

Vertebral sequence	Control group	Experimental group	T	*P*
L1	2.51 ± 2.21	1.07 ± 0.96	2.124	0.176
L2	2.85 ± 2.29	1.42 ± 0.87	2.024	0.185
L3	3.95 ± 2.31	2.40 ± 1.68	1.764	0.214
L4	7.5 ± 7.38	3.01 ± 2.02	2.056	0.182
L5	9.01 ± 3.85	4.81 ± 1.71	5.976	0.035*

*P < 0.05

## Discussion

### Analysis of the results of the X-ray and anatomical specimens

Table 1 demonstrates that in X-ray and anatomical specimens, the angle from the upper to the lower lumbar shows a gradually increasing trend, and the differences among each segment were statistically significant (*P* < 0.05). This is due to the fact that the closer to the sacrum, the greater the angle of the lateral margin of the isthmus and the axis of the pedicle, allowing more attachment to the strong lower lumbar ligamentous structures and bringing them into line with normal biomechanics. Therefore, the author considers that the lumbar vertebrae can be divided into two categories: L1 and L2 can both belong to the upper lumbar; while L3, L4, and L5 can be classified as the lower lumbar. So in screw placement, the upper region can form a right angle, while in lower at a larger angle which is set at approximately 100°.

The differences between the two genders at L1, L2, and L3 were not statistically significant. However, the angles were larger in women than in men at the L4 and L5 segments, the differences were statistically significant, and the deviation was 2–3°. It may be correlated to the difference in the physiological curvature of the lumbosacral region between genders. This suggests that special attention should be paid to assisted the screw placement in L4 and L5 vertebrae by identifying the above anatomical markers in different gender group.

The differences at each segment between the anatomical and imaging measurements were not statistically significant.

### The difference between the lateral margin of isthmus with the lateral margin of lamina and their angles

**Fig. 4:**
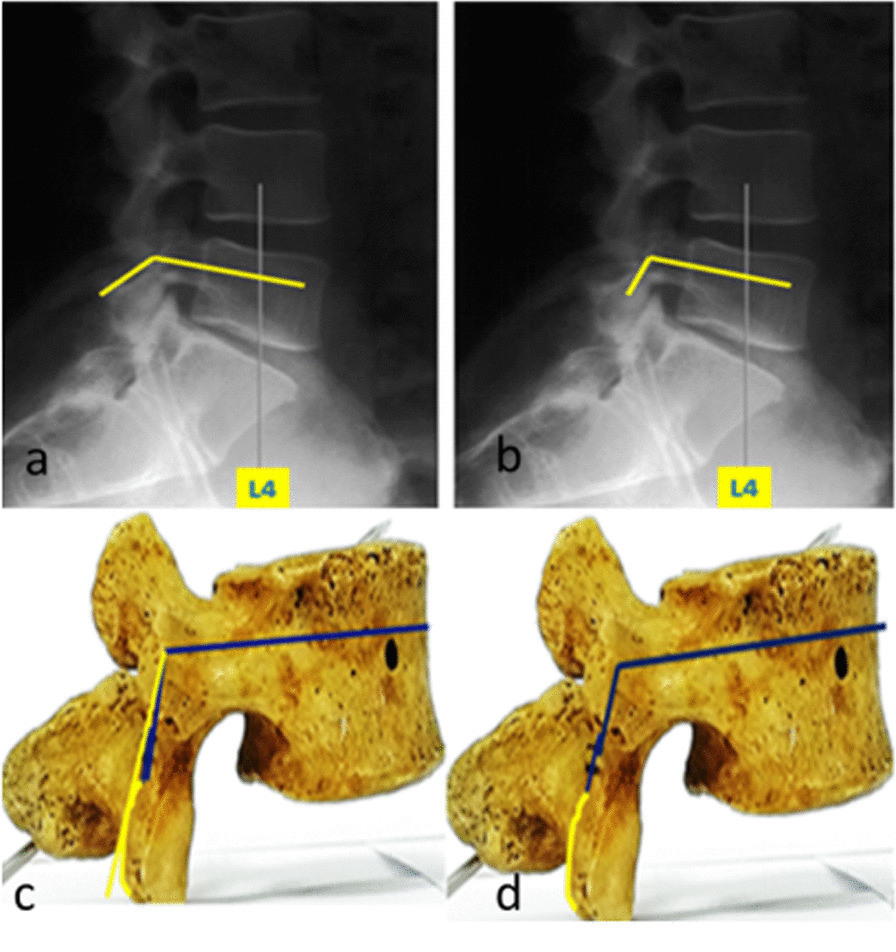
Imaging and anatomical measurement of the angle between the axis of the lumbar pedicle and the lateral margin of the isthmus or lateral margin of the lamina. **a, c** Long yellow line: the angle between the axis of the lumbar pedicle and the lateral margin of the lamina (the line between the lower margins of superior and inferior articular processes in the same segment). **b**, **d** Short blue line: the angle between the axis of the lumbar pedicle and the lateral margin of the isthmus (the line between the superior and inferior articular processes of the same segment)

Figure 4 illustrates the significant difference between them. Because the lateral margin of the isthmus is not affected by the radian of the inferior margin of the lamina and facet joint, the measurement numerical values of its angle between the axis of the pedicle are slightly smaller. If the inferior margin of the lamina or facet joint has severe hyperplasia, the degree of angle between the lateral margin of the lamina and the axis of the pedicle on X-rays would further increase, which is not as constant as the lateral margin of the isthmus mentioned above.

**Fig. 5:**
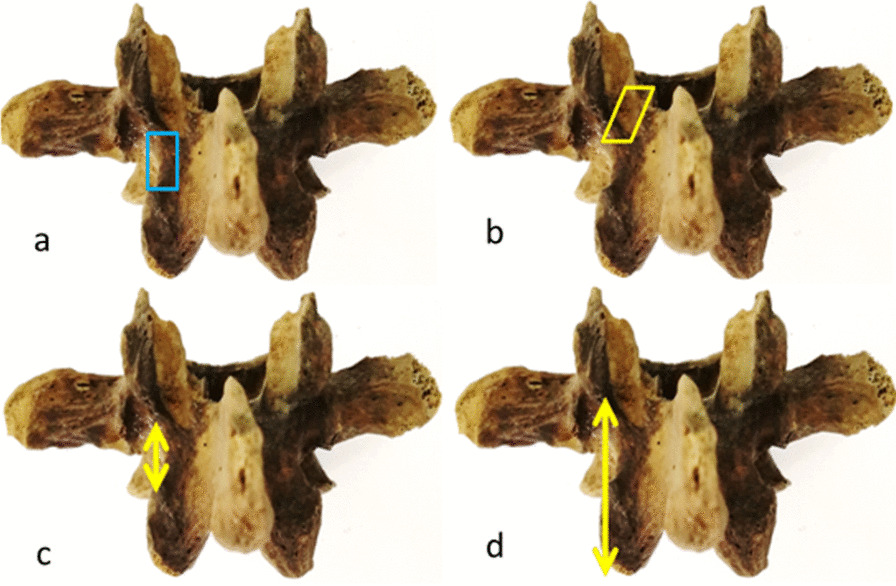
The correlation and difference among the lateral isthmus, the true isthmus, and the lateral margin of the lamina in anatomy. **a** Lateral isthmus; **b** True isthmus; **c** Lateral starting point and endpoints of the isthmus; **d** Starting point and endpoints of the lateral margin of the lamina

The isthmus and lamina are both parts of the vertebral arch, according to the definition. However, these two are not of the same anatomical structure. The isthmus is most likely to be confused with the lamina [10]. The isthmus refers to the junction of the superior and inferior articular processes, while the lamina is derived from the expansion of the bilateral pedicle of the vertebral arch in the backward and inward direction and convergence in the posterior midline. The isthmus is the inherent anatomical landmark of lumbar vertebrae, it includes two different parts [11–13]. Among these, the lateral pars of the isthmus is an osseous pillar that connects the superior and inferior articular processes and is located at the lateral margin of the lamina (Fig. 5a). The true isthmus of the vertebral arch (isthmic pars or pars interarticularis) refers to the location of the isthmus fissure (Fig. 5b), which is the part of the posterior bone structure of the spine located between the pedicle and lamina [11–13]. In the present study, the isthmus that forms an included angle with the axis of the pedicle refers to the lateral isthmus, instead of the true isthmus.

The lateral margin of the lamina is the part between both the lower margins of the unilateral superior and inferior articular processes of the same segment [5, 14] (Fig. 5d). But the lateral pars of the isthmus is the part between the lower margin of the superior articular process and the upper margin of the inferior articular process of the same segment (Fig. 5c). These two anatomic definitions have the same starting point but different ending points.

In anatomical morphology, there is a certain degree of radian between the surface of the lateral margin of the isthmus and the lateral margin of the lower lamina 5 (Figs. 4c, d). The surface of the lateral margin of the isthmus is a straight line (Fig. 4b, d), but the lower margin of the lamina is backward and slightly warps, especially in the lower lumbar L4 and L5 segments (Fig. 4a, c). Other scholars choose the starting and ending point of the lateral margin of the lamina to serve as a benchmark line, this should be correspondingly adjusted according to the degree of back warping of the lamina, especially in the lower lumbar. Moreover, this line cannot be observed in the anatomical specimen or operation. This non-present connecting line or plane can only be imagined by the operator, and based on hypothesis, further infers the angle of the screw in the pedicle, causing a certain deviation.

The anatomical adjacent relationship with the screw entrance point: The isthmus is the nearest and fixed anatomical marker for the vertex of the crista lambdoidal (the junction of the lateral margin of the isthmus and accessory process) which is the insertion point for the screw. Along the straight lateral margin of the isthmus, it is easy for the operator looking for it. These also would enable the measurement of its angle conveniently and accurately. The lamina is infero & internal to the isthmus. It is relatively far and a certain radian from the screw entrance point. Therefore, the author did not consider it is an ideal anatomical reference.

### Clinical application of the angle

The degeneration of the lumbar facet joint has been found in 89.2% of a 60–69 years old population [15–20]. If the connecting line between the lower margins of the superior and inferior articular processes of the same segment was used, the severe degeneration of the articular process would affect the operator in determining the angle.

But the lateral margin of the isthmus will not degenerate, and its angle between the axis of the pedicle in various segments follows a certain rule and does not change with the position of the patient during the operation.

The statistical analysis of the screw placement before and after the junior doctors learned the new method revealed good accuracy (Table 3). The difference in the deviation rate of screw placement before and after the learning was statistically significant only in the L5 segment (*P* < 0.05). This suggests that it is particularly important to learn this method for the screw placement of the L5 vertebrae. In addition, the difference in the overall excellent rate of screw placement was statistically significant (*P* < 0.05). In the upper lumbar, the screw was only required to be perpendicular to the surface of the vertebral plate, which was not very difficult. However, it was relatively difficult to place the screw in the lower lumbar due to the presence of the lumbosacral angle. Furthermore, in clinical practice, the number of patients with degenerative diseases in the lower lumbar is relatively more.

The angle should be accurately measured on the X-ray before the operation, so the accuracy of screw placement can be further improved, operation time is shortened, and the dose of X-ray radiation is reduced.

## Conclusion

The screw placement that refers to the angle between the axis of the pedicle and the lateral margin of the isthmus would not be affected by the degeneration of the facet joint, position, and lumbosacral angle, and can be controlled more accurately.

## Data Availability

The data used to support the findings of this study are included within the article.

## Abbreviations

SSA: Sagittal screw angle
PACS: Picture archiving and communication system
